# Pectate Lyase *FvePL1* Is Required for Pollen Fertility and Mediates Drought Response in Woodland Strawberry

**DOI:** 10.3390/plants14233583

**Published:** 2025-11-24

**Authors:** Xiaolong Huang, Na Li, Guilian Sun, Linfang Zhang, Yuqian Wang, Yu Jiang, Huiqing Yan

**Affiliations:** 1 School of Life Sciences, Guizhou Normal University, Guiyang 550001, China; huangxiaolong@gznu.edu.cn (X.H.); lina@gznu.edu.cn (N.L.); guilian.sun@pku-iaas.edu.cn (G.S.); zhanglinfang@gznu.edu.cn (L.Z.); wangyuqian@gznu.edu.cn (Y.W.); 2 Key Laboratory of Plant Physiology and Development Regulation, Guizhou Normal University, Guiyang 550001, China; 3 Key Laboratory of State Forestry Administration on Biodiversity Conservation in Mountainous Karst Area of Southwestern China, Guizhou Normal University, Guiyang 550001, China

**Keywords:** *Fragaria vesca*, pectate lyase, pollen, achene, drought

## Abstract

Successful fertilization is essential for fruit bearing and yield enhancement, relying on male gametophyte which facilitates sexual reproduction by transferring the sperm cell to the ovule. To accomplish this task, pectate lyase is secreted to lubricate the sperm cell towards the female partner by different strategies. However, the specific impact of strawberry PL in male sterility and achene development remained elusive. Here, we systematically investigated the functions of diploid strawberry *Fragaria vesca pectate lyase 1* (*FvePL1*), determining its localization in the cell wall and membrane. In situ hybridization presented its maximum expression in the anther, particularly the endothecium, connective tissue, and septum. Analysis of RNAi mutants and green fluorescent protein (GFP)-tagged overexpression lines demonstrated that the failure of *FvePL1* significantly inhibited the fruit set due to stunted achenes. In addition, the deficiency of *FvePL1* expression resulted in a 68.29% reduction in the number of pollen grains, a 73.27% decrease in pollen viability, morphological alterations of exine and intine, impaired pollen tube, and the inability of the sperm nucleus to reach its target due to the delayed and incomplete tapetal degeneration. In addition, the suppression of *FvePL1* resulted in a 65.02% increase in survival rate withholding irrigation for 30 days, conferring enhanced drought tolerance by negatively influencing cell wall structure. Therefore, this study identified *FvePL1* as a crucial regulator of pollen development, fertilization, and achene maturation and abiotic stress. These findings provide a framework for advancing research on the development of the male gametophyte in strawberry and even yield optimization in Rosaceous crops.

## 1. Introduction

The emergence of fruits marks an evolutionary breakthrough, profoundly enhancing seed dispersal efficiency and initiating the adaptive radiation of angiosperms. A successful fruit set necessitates precise pollination via pollen transfer to the pistil. Upon successful fertilization, the ovary develops into a fruit, sheltering embryonic seeds destined for germination for the next generation of flowering plants [1]. However, successful fertilization requires normal male gametophyte, indispensable for orchestrating the biological functions of the elaborately structured pollen grain [2]. Pollen development within the anther locules of the stamen involves a multistage process requiring precise orchestration [3]. In addition, the delivery of sperm cells to the female gametophyte was facilitated by pollen tube extension from the grain’s apex through the transmitting tract’s extracellular matrix, culminating in stigmatic papillae penetration and subsequent access to maternal reproductive tissues [4]. During this process, the cell wall is crucial for directional expansion of pollen tube germination and the success of double fertilization [5].

Pectin, as a natural macromolecule in biomedical and drug delivery applications, is a principal macromolecular polysaccharide component of the growing pollen tube apex and has been reported to be indispensable for fertilization [6,7]. Its remodeling is governed by wall-modifying enzymes that balance pectin depolymerization and polysaccharide deposition, establishing a dynamic equilibrium of elastic, plastic, and rigid properties necessary for tube elongation [8,9]. Investigations of pectin participate into pollen cell walls have been elucidated in *Arabidopsis* [8]. Such architectural complexity involving pectin underscores the capacity of pollen tube to overcome the mechanical and biochemical barriers of pistil [10]. Pectate lyases (PLs) have emerged as critical regulators of pollen tube growth and fertilization in *Arabidopsis*, notwithstanding the significance of pectin modification to regulate cell wall stability and extensibility [11,12]. They are accountable for the degradation of pectin by cleaving the α-1,4-polygalactyronic acid chain and its activity is stringently regulated to ensure proper cell wall assembly and remodeling during diverse developmental processes [13]. Additionally, *Arabidopsis* PLs have been demonstrated to have maximal expression in the mature pollen grains and post-pollination pistils, underscoring their indispensable roles in pollen tube elongation and fertilization [14]. Recently, they have been proved to be indispensable for facilitating the loosening of the innermost layer of the pollen wall, which is crucial during the initial pollen hydration and pollen tube emergence loosening during the initial stages of pollen tube germination in *Arabidopsis* [15]. Moreover, PLs are secreted by the elongating pollen tube into the extracellular matrix, and enable modification of transmitting tract pectin networks, enhancing pollen tube penetration through pistil tissues to expedite pollen tube growth and to aid the sperm cell in reaching the ovule in *Arabidopsis* [15].

Strawberry serves as an excellent model for investigating fertilization-induced fruit development, owing to its unique fleshy fruit derived from a receptacle, an enlarged stem tip supporting hundreds of seed-bearing ovaries [3]. Fertilization triggers the production of fertilized achenes, with fruit size and yield demonstrating a positive correlation to seed number. The removal of fertilized ovules from the receptacle prevents the formation of fleshy fruit, underscoring that insufficient male gametophyte compromises fruit set and reduces yield. Generally, more achenes promoted larger and more bountiful fleshy fruits. Therefore, the regulatory mechanisms governing pollen development are requisite for enhancing fruit production [16]. Sixteen *FvePL* homologs have been identified in the diploid woodland strawberry *Fragaria vesca* [17]. Co-expression network analysis revealed that *FvePLs* had close association with genes involved in the development of vegetive and reproductive organs. In addition, histological observations demonstrated that *FvePL1*, *4* and *7* enhanced cortical cell division and expansion, thereby promoting fruit softening, also in cultivated strawberry [18]. It has been acknowledged that the decreased expression of *FvePL1* (*FvH4_2g19540*) was linked to altered petal architectures, and significantly reduced the number of pollen grains [17]. Nevertheless, no experimental evidence has established its involvement in pollen tube morphogenesis via direct cell wall remodeling. Therefore, there is a necessity to comprehend how *FvePL1* guides the pollen and fruit maturation at the cellular and subcellular levels.

In this work, we aimed to elucidate the function of *FvePL1* in pollen tube growth, specifically testing its essential roles in cell wall remodeling and the biomechanical properties governing this process. Through an integrated approach combining physiological, genetic, and cytological analyses, we demonstrate that *FvePL1* is critical for pollen fertility, achene development and enhanced drought tolerance. Our findings provide a molecular mechanism for male gametophyte development, offering a strategic target for enhancing strawberry fertility and yield through genetic breeding.

## 2. Results

### 2.1. Molecular Characterization of FvePL1 Which Was Maximumly Expressed During Anther Maturation and Fruit Transformation

**Figure 1:**
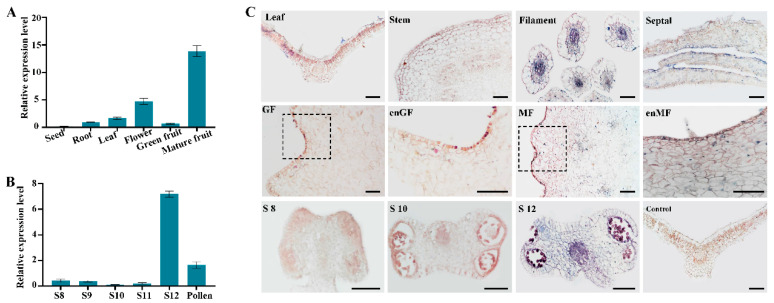
Characterization and spatiotemporal dynamics of *FvePL1*. (**A**) Tissue-specific and (**B**) developmental expression profiling of *FvePL1* in anther ranging from different stages, including leaf, stem, filament, septal, S8 (developmental stage 8 of anther), S10 (developmental stage 10 of anther), S12 (developmental stage 12 of anther), green and mature fruits; (**C**) in situ hybridization analysis of *FvePL1*. GF, green fruit; MF, mature fruit; enGF and enMF, enlarged image of black box in green fruit and mature fruits, individually, bar = 100 μm.

**Figure A1:**
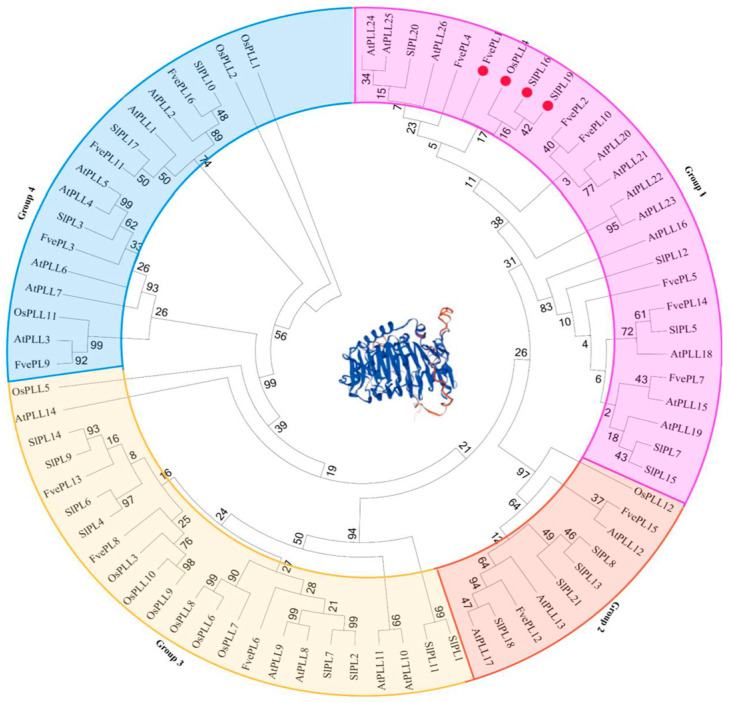
Phylogenetic analysis of FvePL1 homologs from *Solanum lycopersicum*, *Oryza sativa*, and *Arabidopsis*. The central micrograph depicted the three-dimensional protein structure of FvePL1. The red dots indicated the homologs to FvePL1.

The coding sequence (CDS) of *FvePL1* is 1341 bp long, encoding 446 amino acids (3 bp is a stop codon) with a molecular mass of 49.81 kD and an isoelectric point of 8.44. To identify proteins homologous to *FvePL1*, a phylogenetic tree was constructed within PL family members from other species, including *Oryza sativa*, *Solanum lycopersicum*, and Arabidopsis, revealing notable sequence similarity to *OsPLL4*, *SlPLL16*, and *SlPLL19*, suggesting conserved functional attributes among these orthologs (Figure A1). The expression profiles of *FvePL1* across six major organs, anthers (at developmental stages of eight, ten and twelve) and mature pollens were detected [3]. The data showed that *FvePL1* was widely expressed in multiple tissues. However, its transcripts were found the most in mature fruit, followed by floral tissues and leaf, with pronounced expression localized to anther stage 12 and mature pollen (Figure 1A,B).

Histological observation further corroborated the transcripts of *FvePL1* in various tissues. Visible signatures of *FvePL1* were observed in epidermal cells and mesophyll of leaves by in situ hybridization. Similarly, they were displayed in epidermal cell layer and parenchyma of stem, filament, and petal, aligning with critical vascular differentiation processes during vegetative organ maturation. Distinct hybrid signals of *FvePL1* were also observed in mature fruit, and the enlarged image of mature fruit confirmed the strong staining in the epidermal cell layer and parenchyma of the cortex cells, suggesting its higher expression at the turning stage to regulate fruit firmness due to degrading pectin. By contrast, there was only weak expression of *FvePL1* in the epidermal of green fruit. No discernable signals were found in anther stage 8 when microspore mother cells appeared and four locules became distinct, and stage 10 when tapetum degeneration was initiated. Conversely, prominent and intense hybridization signals of *FvePL1* were noticed in the endothecium, connected tissues of anthers, epidermis and pollen, suggesting its potential roles in degenerating middle layers, thus facilitating the degradation of the primary cell wall and promotion of pollen penetration (Figure 1C).

### 2.2. FvePL1 Was Localized in Cell Wall, Cell Membrane and Cytoplasm

**Figure 2:**
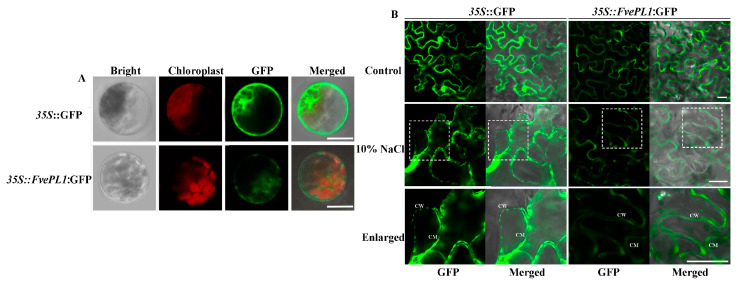
Subcellular localization of FvePL1. (**A**) Fluorescence microscopy analysis in transiently expressed Arabidopsis protoplast cells. Overlay of the GFP fluorescence (green), chlorophyll autofluorescence (red), bright field, and the combined images, bar = 10 μm. (**B**) *FvePL1*:GFP subcellular localization in epidermal cells of *N. benthamiana* leaves under control conditions and plasmolysis (10% NaCl). The white boxes were enlarged as shown below. CW, cell wall; CM, cell membrane, bar = 20 μm.

To explore the specific localization of FvePL1 in cells, the vector of 35S::*FvePL1*:GFP was introduced into Arabidopsis protoplasts for subcellular localization (Figure 2A). Microscopic visualization showed that robust green fluorescent signals, as the positive control of 35S::GFP-transformed protoplasts, were localized in the whole cell. Meanwhile, FvePL1 was predominantly presented in the cell membrane and cytoplasm. To further clarify whether FvePL1 was localized in the cell wall, 35S::*FvePL1*:GFP and control were individually transiently transformed into *Nicotiana benthamiana* leaves following plasmolysis induction via 10% NaCl treatment. While non-induced controls exhibited uniform fluorescence throughout the cellular compartment, plasmolyzed tissues expressing 35S::GFP displayed distinct GFP fluorescence localized to the cell wall (Figure 2B). When the cells expressed 35S::*FvePL1*:GFP after plasmolysis, the enlarged image shows that the strong green signals were found to be localized in the cell walls in plasmolyzed cells, implicating *FvePL1* in dynamic cell wall modulation.

### 2.3. Reduction in Fvepl1 Resulted in Fruit Defects and Lower Seed Production

**Figure 3:**
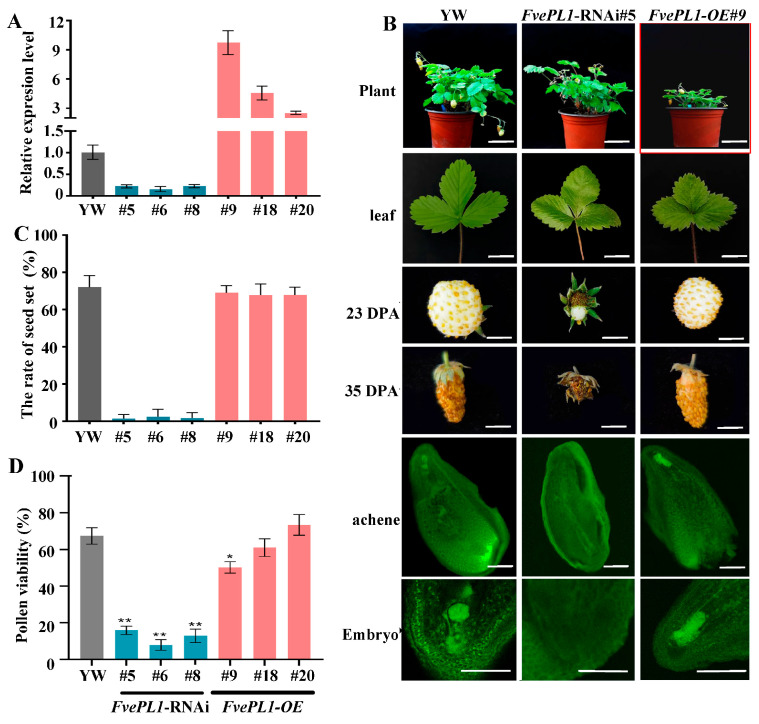
Phenotypic characterization of *FvePL1* transgenic strawberry plant. (**A**). Relative expression level of *FvePL1*, (**B**). Morphological comparisons of plants (bar = 5 cm), leaf (bar = 2 cm), fruits at 23 days post-anthesis (DPA), 35 DPA (bar = 0.5 cm), achene and embryo (bar = 100 μm), (**C**). Seed rate, (**D**). Pollen viability, in YW and transgenic lines. Statistical analysis was performed using one-way ANOVA using Tukey’s test (*, *p* < 0.05, **, *p* < 0.01).

To analyze detailed functions of the *FvePL1*, an over-expression (OE) and RNAi vectors were constructed and transformed into YW5AF7 (YW) genetic background via agrobacterium-mediated method. After hygromycin resistance screening, transgenic seedlings were further determined using qRT-PCR to test the transcript levels of FvePL1 to identify the positive lines (Figure 3A). The upregulation of expression was found in *FvePL1-OE* #9, #18 and #20, which were a 9.18-fold, 4,82-fold and 2.89-fold increase compared to that of the YW, individually. By contrast, the expression levels were remarkably lower in *FvePL1-RNAi#5*, #6 and #8 compared to control. Thus, seedlings of *FvePL1-OE#9* and *FvePL1*-RNAi*#5* were further subjected to comprehensive phenotypic analysis compared to YW.

Morphological analysis of whole plants showed that *FvePL1-OE* displayed wrinkled leaves, smaller seedlings and stunted growth compared to YW and RNAi counterparts. Fruits of RNAi lines did not develop further after pollination at 23 and 35 days post-anthesis (DPA) observed using stereoscopic analysis (Figure 3B). Notably, *FvePL1* downregulation directly correlated with impaired fruit set and seed development. With deep observation using confocal laser scanning microscopy, a normal embryo could be detected at the top of the ovule, and the central cells also differentiated normally to form endosperm in *FvePL1-OE* and YW plants, revealing marked differences (Figure 3B). However, the images show that a tubular structure full of dead cells and premature endosperm cellularization was present in the ovule of *FvePL1*-RNAi, resulting in the abortion of forming normal embryos and endosperm. No discernable difference was noticed in seed rate in YW and overexpression lines, which were both approximately 75%, whereas they were remarkably decreased in RNAi lines which were only 2%~4%, in correspondence with the reduction in fruit bear and the lower seed set attributed to the downregulation of *FvePL1* (Figure 3C). In addition, pollen viability in the *FvePL1*-RNAi line were individually 19.24%, 12.76% and 18.67%, whereas the values in overexpression lines were 53.84%, 61.16% and 74.58%, resulting in a 73.27% reduction (Figure 3D).

### 2.4. FvePL1 Is Required for the Development of Exine and Intine of Pollen Grains

**Figure 4:**
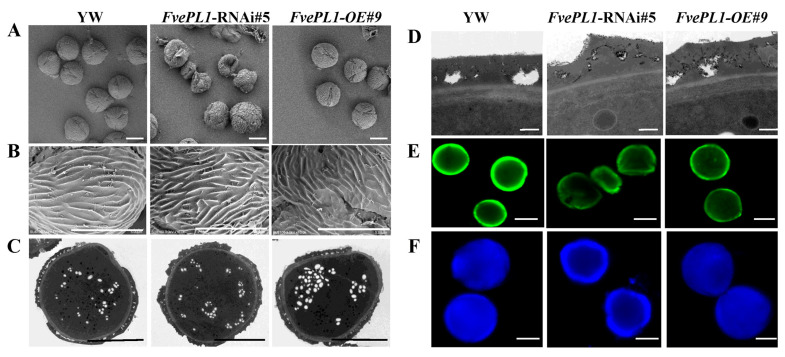
Aberrant pollen cell wall development in *FvePL1*-deficient plants. (**A**). Scanning electron microscopy (SEM) images of mature pollen, (**B**). Surface enlarged morphology, (**C**). Transmission electron microscopy (TEM) observation of mature pollen, (**D**). The enlarged images of the pollen wall structure, (**E**). Cryosections of pollen grains stained by auramine O staining with a strong fluorescence of the exine, (**F**). Calcofluor white fluorescence of the inner cellulosic sublayer of the intine of three genotypes. Bar = 10 μm.

To assess the influence of *FvePL1* on pollen cell walls, scanning electron microscopy (SEM) was utilized to investigate the ultrastructural changes in pollen grains and adjacent cell walls regulated by *FvePL1* (Figure 4A). The pollen surface of YW or *FvePL1-OE* has multiple germination pores, accompanied by orderly arrays of raised striations on the surface of pollen grains (Figure 4B). In contrast, most *FvePL1*-RNAi pollen grains displayed irregular contours, deformed convex lines and depressed inward, resulting in abnormal aperture, and ultimately repressing pollen germination (Figure 4B).

To provide more detailed information on the internal morphology of pollen grains at the ultrastructural level, mature pollens from the new open flowers of three genotypes were fixed and processed to prepare for transmission electron microscopy (Figure 4C). The fine exine was composed of the baculum and the tectum. Except for more dispersed starch granules found after repressing *FvePL1*, no apparent abnormalities were observed in the cytoplasm. However, there was significantly different morphology of intine and exine regulated by *FvePL1* through enlarging the cell wall structures (Figure 4D). First, the intine thickness of pollen became thicker in *FvePL1*-RNAi lines. Secondly, less white signals were present around the intine, indicating that fewer starch granules were accumulated in the exine after the deficiency of *FvePL1*. Thirdly, the surface of exine in *FvePL1*-RNAi lines, especially the structure of tectum, were much more overflowing furrow regions, whereas the smooth surface of exine was observed in YW. By focusing on the structural characteristics of the exine and intine layers, we identified the roles of *FvePL1* in the modifications of the cell wall architecture.

Exine is mainly composed of sporopollenin and cellulose to protect the internal structure of pollen from damage. Intine with a pectocellulosic composition is thicker under the pollen apertures, from where the pollen tube emerges. Auramine O staining was used to test the functions of *FvePL1* on the exine using confocal microscopy (Figure 4E). The images illustrate that strong and continuous green fluorescence was observed in the outline of YW and *FvePL1-OE*. On the contrary, the fluorescence of *FvePL1*-RNAi plants was discontinuous and weak in the outer wall boundary. We further used Calcofluor white to examine the intine influenced by *FvePL1* (Figure 4F). Consistently, a hollow center and only weak blue fluorescence of the outline were noticed in pollen grains of *FvePL1*-RNAi, while substantial blue signals were found in YW and *FvePL1-OE*. The abnormal development of exine and intine demonstrated that *FvePL1* dysregulated exine and intine organization, precipitating pollen developmental defects.

### 2.5. FvePL1-RNAi Lead to the Decreased Pollen Number Due to Degradation of Tapetum

**Figure 5:**
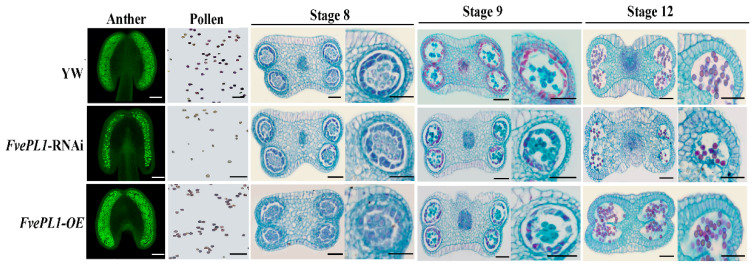
The number of pollen grains were impaired in *FvePL1*-RNAi lines. The laser confocal microscopy images of pollen grains in the anther of three genotypes on the first day post flowering. In addition, the cytological observation of the anther at stage 8, 9 and 12 in YW, *FvePL1*-RNAi and *FvePL1-OE* lines were detected and the images zoomed. Bar = 100 μm.

To determine the functional role of *FvePL1* in pollen development, anthers at day 1 post-anthesis were harvested and analyzed via confocal microscopy. The images showed that *FvePL1-OE* exhibited pollen quantities equivalent to YW, whereas a statistically significant reduction in mature pollen grain of *FvePL1*-RNAi compared to other two genotypes (Figure 5). To elucidate the mechanism underlying the diminished pollen production, developmental dynamics across anther maturation were examined through paraffin sectioning. Cytological analysis demonstrated that all genotypes achieved normal anther dehiscence by stage 8, with no discernible differences in microsporogenesis initiation, including synchronized meiotic entry of tetrads or four-locule compartmentalization. Subsequent histological evaluations revealed progressive cellular differentiation: locular expansion facilitated tetrad release by stage 9, concurrent with middle layer degeneration, while tapetal cells in wild-type and *FvePL1-OE* lines underwent complete degradation by this phase. However, the failure degradation of tapetum in *FvePL1*-RNAi was noticed, resulting in no distinct separation at Stage 12. In addition, more round and uniformly larger pollen grains were formed in YW and *FvePL1-OE*. On the contrary, limited viable pollen grains were differentiated, suggesting the aborted pollen grains caused by the downregulation of *FvePL1*.

### 2.6. FvePL1 Is Essential for Pollen Germination

**Figure 6:**
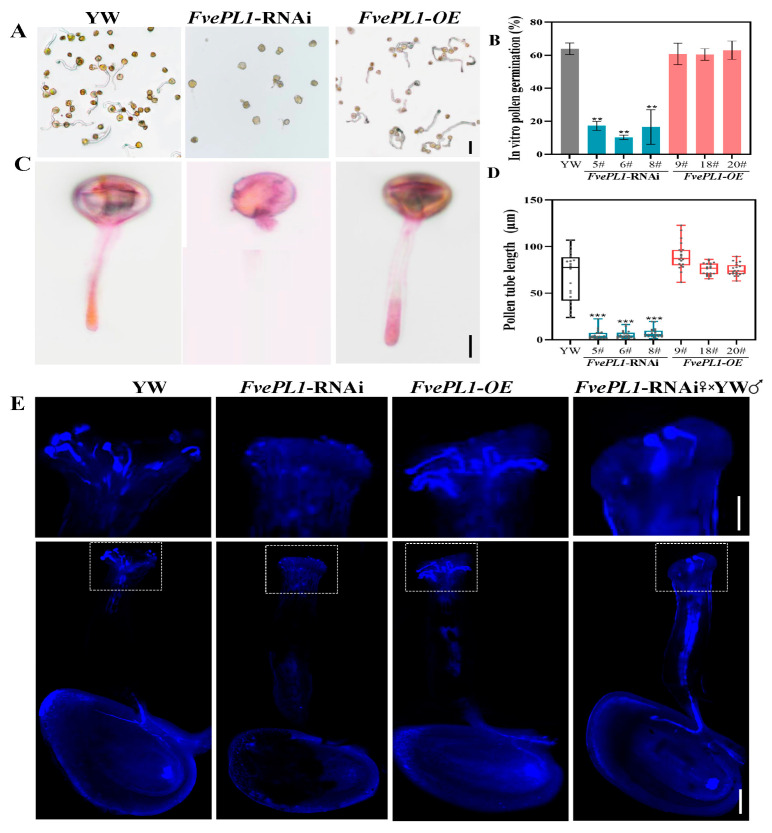
Pollen germination was significantly impaired in *FvePL1*-RNAi plants. (**A**). Morphological characterization of pollen germination, bar = 20 μm. (**B**). Comparison of pollen germination percentages in vitro. (**C**). Histological observation of germinated pollen tube, bar = 20 μm. (**D**). The length of pollen tube among overexpression, RNAi of *FvePL1* and YW. The data were the average values of each of the three lines with comparison between multiple samples determined by one-way ANOVA using Tukey’s test (**, *p* < 0.01; ***, *p* < 0.001). (**E**). In vivo pollen-tube growth in pistils in 20 min after pollination of YW, *FvePL1*-RNAi, *FvePL1-OE*, and reciprocal pollination experiments and zoomed images. Bar = 50 μm.

To assess the influence of *FvePL1* on pollen germination, we observed pollen germination by an in vitro germination experiment (Figure 6A). The results showed that the most mature pollen grains of YW and *FvePL1-OE* transgenic plants uniformly germinated and an opaque pollen tube emerged from pollen grains. Differently, in addition to the smaller number of pollen grains in *FvePL1*-RNAi lines, barely any pollen tube could be observed. In addition, we found that the pollen germination rates of YW and *FvePL1-OE* transgenic lines were individually 61.47%, 60.98% and 66.79%. However, there was no more than 20% after disrupting the *FvePL1* expression, which decreases by 68.29%, in accordance with the results of pollen viability (Figure 6B). Despite some pollen tube produced in *FvePL1*-RNAi lines, the tube length was terrifically shorter than 10 μm by staining observation (Figure 6C). No doubt, the pollen tube lengths in *FvePL1-OE* and YW were both longer than 40 μm, especially up to 95 μm in YW (Figure 6D). These findings collectively demonstrate that *FvePL1* deficiency disrupts pollen germination and severely impairs tube elongation.

To further detect the in vivo germination of mature pollen, we collected pistils after pollination and stained pistils utilizing aniline blue (Figure 6E). The images showed obvious outlines and distinct blue fluorescence of the transmitting tract and ovary in *FvePL1-OE*, which were no apparent abnormalities from YW, indicating the male sperm cell could successfully move their target after pollination (Figure 6E). By highlighting their stigmas, clear long pegs of germination tubes were displayed, especially in the *FvePL1-OE* line. In contrast, only slight blue signals were found in *FvePL1*-RNAi lines, indicative of no obvious pollen observed on stigma. When further zooming in the top of pistils, barely no germination tube of *FvePL1*-RNAi was photographed, consistently with the results of in vitro germination experiments, corroborating that the decrease in *FvePL1* reduced the pollen germination rate and inhibited pollen tube elongation. Notably, reciprocal pollination experiments confirmed retained female fertility in *FvePL1*-RNAi plants, as wild-type pollen exhibited normal germination and tube extension within mutant pistils, indicating the normal female fertility of *FvePL1*-RNAi which could receive pollen of YW normally. Based on in vitro and in vivo germination experiments, despite the normal development of stigma and female gametophyte, the reduction in FvePL1 resulted in abnormal pollen germination, significantly shortening the length of pollen tubes.

### 2.7. Downregulation of FvePL1 Improved Drought Tolerance

**Figure 7:**
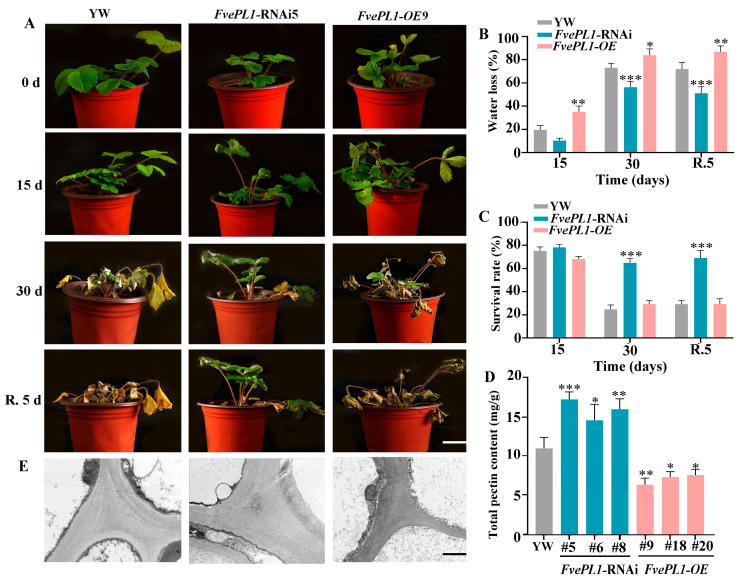
Downregulation of *FvePL1*-enhanced drought tolerance. (**A**). Phenotypic assessments of three genotypes subjected to 15 d and 30 d drought stress, as well as R. 5 d (recovery 5 d) (bar = 5 cm). (**B**). Water loss rate, (**C**). Survival rate and (**D**) total pectin content in YW and *FvePL1* transgenic plants. The asterisks represent the significant differences between YW and *FvePL1* transgenic plants. Statistical analysis was performed using one-way ANOVA using Tukey’s test (*, *p* < 0.05; **, *p* < 0.01; ***, *p* < 0.001). (**E**). Ultrastructural analyses of cell corners of leaf epidermis were analyzed (bar = 2 μm).

To elucidate the role of *FvePL1* in drought stress, three genotypes underwent water deficit treatment. Phenotypic observation revealed that YW exhibited subtle leaf hyponasty, a hallmark of moderate water deficit at 15 days. In the same period, *FvePL1*-*OE* plants displayed pronounced drought-induced symptoms, including downward leaf curvature, whereas *FvePL1*-RNAi plants maintained significantly normal morphology compared to other counterparts under identical conditions. Prolonged drought to 30 days exacerbated overexpression of plant stress symptoms, manifesting severe stress symptoms, including leaf desiccation and whole-plant wilting. By contrast, RNAi plants exhibited markedly attenuated drought symptoms, corroborating drought tolerance after *FvePL1* suppression. After 5 d recovery, plants of *FvePL1*-RNAi resumed normal growth (Figure 7A). Evaluations of water loss and survival rates indicated a pronounced enhancement in drought tolerance for *FvePL1* RNAi plants relative to both YW and overexpression lines. This was particularly evidenced by a 55.04% increase in survival rate following a 30-day period of irrigation withholding (Figure 7B,C). Furthermore, in contrast, *FvePL1*-OE displayed a reduced drought tolerance and lower survival rate. In addition, the total pectin accumulation of *FvePL1* RNAi was almost 1.52- and 2.47-fold than that in YW and *FvePL1*-OE (Figure 7D). Correspondingly, ultrastructural analysis of cell wall architecture revealed striking morphological divergence. The electron-dense material observed in the tricellular junction zone of *FvePL1*-RNAi was more intensive than other two genotypes (Figure 7E). By contrast, the adjacent cells of *FvePL1-OE* were separated, and the junction zone was degraded, loose and separated.

## 3. Discussion

In this study, we highlighted the intricate relationship between *FvePL1* and male reproductive tissues. A noticeable gap in the current research is the exploration of *FvePLs*’ broader roles beyond pectin modification, potentially interacting with features of pollen grains and functions in reproductive plant tissues.

### 3.1. FvePL1 Implicated in Male Gametophyte Development Is Indispensable for Fruit Set

FvePL1, harboring a conserved pectin lyase domain, was localized to the cell wall and membrane. In addition, in situ hybridization revealed its predominant expression in the endothecium, connective, septum, epidermal cell layers and parenchyma of the cortex cells. Transgenic analyses demonstrated that *FvePL1* deficiency caused severe fruiting failure, evidenced by defective and stunted achenes. By a comprehensive assessment of the entire biological process of male gametophyte, the downregulated *FvePL1* led to the decreased number of pollen grains, less viability, morphological aberrations in exine and intine, impaired pollen germination germinate, defected formation of pollen tubes, and ultimately led to failed fertilization. All the data illustrated that *FvePL1* played robust and vital roles in the male sterility of strawberry, aligning with most functions of *PL*-like in Arabidopsis [18,19], *Brassica campestris* [20], and *Gossypium hirsutum* [21]. Therefore, *FvePL1* is required for developing achene and strawberry yield [18].

### 3.2. The Aborted Pollens Caused by the Reduction in Fvepl1 Initiated from the Incomplete and Delayed Degradation of Tapetum and Septum

To explore the mechanism underlying the function of *FvePL1* on the male gametophyte development, our findings discovered that *FvePL1* suppression disrupted the development of tapetum and pollen wall biogenesis, leading to male sterility defects. Cytological observation revealed conserved microsporogenesis stages across three genotypes. Tetrads were held and microspore mother cells tightly packed within the anther locules before entering meiosis. Interestingly, YW and overexpression lines exhibited synchronized locule expansion, allowing individual tetrads to separate from each other. In contrast, the incomplete and delayed middle layer degradation was noticed in the *FvePL1*-RNAi lines, suggesting that the failure of pollen development was initiated form tapetum and septum degradation. The tapetum has been recognized to supply nutrients and metabolic substances to aid pollen grain maturation. It undergoes programmed cell death (PCD) and secretes enzymes that degrade cellular organelles. This degradation process helps in the formation of sporopollenin which is essential for exine formation, and aids the release of mature pollen grains from the anther [22].

The incomplete and delayed degradation of tapetum and septum caused by the downregulation of *FvePL1* can be described as follows: (1) *FvePL1* contributed in disrupting the integrity of the cell wall of tapetum; (2) *FvePL1* might participate in the supplied process of nutrients from tapetum; and (3) *FvePL1* affected the pectin-mediated signaling cascades pathway that exhibited premature PCD of tapetum and disordered cell wall. Cell death has been hypothesized to be necessary to provide nutrients to growing pollen tubes but also to facilitate the pollen tube penetration [4]. These coordinated processes ensure synchronized pollen development, whereas dysregulation induces developmental discordance. Collectively, *FvePL1* acts as a negative regulator of tapetal and septal degradation pathways, where its diminished activity disrupts reproductive efficiency and strawberry yield.

### 3.3. Different Regulatory Mechanisms of FvePL1 Influencing Pollen and Fruit Development

Despite its downregulation inducing severe male sterility defects, the overexpression of *FvePL1* presented no discernable difference in pollen maturation phenotypes compared to YW. Integrated analysis of in vitro and in vivo germination indicated that *FvePL1* deficiency selectively disrupted pollen germination and tube elongation, despite maintaining pistil integrity and female gametophyte functionality. This dysfunction likely arises from perturbed signaling pathways essential for pollen tube biogenesis, independent of post-germination pollen–pistil interactions [23]. In contrast to pollen defects, *FvePL1-OE* transgenic lines accelerated fruit softening relative to wild-type controls [17]. The divergent phenotypes caused by *FvePL1* elicited a tissue-dependent regulatory molecular mechanism. The digested pectin could act as signaling molecules to feedback control of tapetum degradation and pollen maturation that is yet to be confirmed [24]. Considering that normal female fertility in the *FvePL1*-RNAi pollen pollinated lines, the fruit-initiated stage of *FvePL1-OE* plants paralleled wild-type progression. With the maturation of fleshy fruit, additional transcription factors might regulate the functions of *FvePL1*. For example, overexpression of *FvePL4* promotes fruit softening due to the ABA-induced FvWRKY48 bound to the *FvePL4* promoter via a W-box [25]. Thus, different transcriptional factors could influence *FvePL1* in regulating pollen and fruit development.

### 3.4. FvePL1 Negatively Mediated Drought Response in Woodland Strawberry

Given that *FvePL1* modulates pectin in pollen which constitutes a heterogeneous, branched, and highly hydrated polysaccharide network within the plant cell wall, it confers enhanced tolerance to drought and osmotic stress [26]. Consequently, the pectin-composed cell wall plays a direct role in the drought stress response [27]. Furthermore, its derivatives, including O-acetylated pectin, regulate growth and biotic/abiotic stress adaptation [28]. Functional analysis of *FvePL1* in strawberry revealed that its overexpression elevated plant dwarfing and disrupted cell wall architecture, especially cell junction zones, thereby decreasing drought tolerance. Conversely, the failure of *FvePL1* expression profoundly increased drought stress. This evidence suggested that pectate lyase modulates cell wall biomechanics and osmotic adjustment through targeted catalysis of alpha-1,4-glycosidic linkages in pectic polysaccharides [29]. Likewise, over-expression of the *PMEI* enhances drought tolerance of *Glycyrrhiza uralensis* [30]. Furthermore, previous studies showed that candidate genes related to cell wall remodeling enzymes (such as PL, PME, PMEI, and expansins) were related with plant response to drought tolerance and could provide an interesting point for developing molecular markers for breeding drought-resistant plants [31]. The findings offer a comprehensive understanding of the contribution of *FvePL1* on pollen development and abiotic stress. *FvePL1* might act in a direct or signal pathway, setting the stage for future research into its deeper mechanism. As climate unpredictability intensifies and challenges for pollen viability and seedling growth emerge, this research shows significance, as *FvePL1* could regulate fertilization efficiency or response to stress.

## 4. Materials and Methods

### 4.1. Plant and Growth Conditions

The *Fragaria vesca* strain Yellow Wonder 5AF7 (YW5AF7) inbred line, derived from the seventh generation of selfing, served as the wild-type genotype in this study. RNA interference (RNAi) and overexpression constructs were both generated in YW5AF7. Seedlings were cultivated in a controlled growth chamber with a continuous 16 h light/8 h dark photoperiod at 22 °C, 65% relative humidity, and 350 μmol m^−2^ s^−1^ light intensity.

### 4.2. Phylogenetic Tree Construction

Protein sequences of FvePL1 were downloaded from the Genome Database for Rosaceae (GDR: www.rosaceae.org/ (accessed on 14 October 2025)) and the homology search was conducted using the amino acid sequences for FvePL1 from the UniProt database (https://www.uniprot.org/ (accessed on 14 October 2025)). Multiple alignment was performed using ClustalW version 2.0 (http://www.clustal.org/clustal2/ (accessed on 14 October 2025)) and the relative accession number list of PL gene family in *Oryza sativa*, *Arabidopsis*, *Solanum lycopersicum*, and *F. vesca* were listed in Table A1. A phylogenetic tree was constructed using the maximum likelihood (ML) method with 1000 bootstrap replicates via MEGA7.0 (http://www.megasoftware.net (accessed on 14 October 2025)).

### 4.3. Subcellular Location

The *FvePL1* coding sequence was PCR-amplified and cloned into the *Xba*I site of the pM999 vector to generate CaMV *35S*::*FvePL1*:GFP plasmid. Protoplasts were isolated from the mesophyll tissues of 4-week-old *Arabidopsis* leaves (Columbia-0 ecotype). A total of 100 μL (2 × 10^4^) protoplast was mixed with 10 μL (10~20 μg) recombinant or control plasmid, then 110 μL PEG solution was added for transfection. Transfected protoplasts were imaged using a ZEISS LSM 710 confocal microscope (Zeiss, Oberkochen, Germany) under a 488 nm blue light laser.

### 4.4. In Situ Hybridization

For cytological observation of *FvePL1* expression, leaf, stem, filament, petal, anther (at developmental stages 8, 10 and 12) [3], green and mature fruits were collected and fixed in RNase-free FAA solution (4% formaldehyde (*v*/*v*), 10% acetic acid, and 50% ethanol), followed by dehydration through graded ethanol series, embedded in paraffin wax and sectioned (8 μm) using Leica RM2255 (Leica, Wetzlar, Germany). A gene-specific cDNA fragment of *FvePL1* was amplified using ISH-F/R primer for in situ hybridization. Their PCR product was then cloned into the pGEM-T vector. Sense and antisense RNA probes were synthesized using SP6 and T7 RNA polymerase, respectively. In situ hybridization experiments were performed, including prehybridization, hybridization, and washing using a DIG RNA labeling Kit (Roche, Basel, Switzerland) applied to the tissue paraffin section [17]. Sides were photographed under a BX53 microscope (Olympus, Tokyo, Japan).

### 4.5. RNAi and Overexpression Constructs and Transgenic Strawberry Generation

For RNAi vectors, the partial coding sequences about 500 bp targeting the at 5′-end coding regions of *FvePL1* were amplified, respectively, by RT-PCR and placed upstream and downstream of pDS1301 in forward directions using *Kpn*I/*Bam*HI and reverse directions using *Spe*I/*Sac*I. To generate the *FvePL1* overexpression, regions of *FvePL1*-GFP were amplified from pM999-*FvePL1*-GFP and cloned into pMDC32 binary vector at the digestion sites of *Kpn*I and *Pac*I which the GFP reporter fused at the C-terminus of the gene. The constructs were individually transformed into *agrobacterium* strain GV3101. *F. vesca* transformation and regeneration were performed using previously published protocols [16]. To confirm transgenic lines, seedlings of various genotypes were sampled. All primers used in this study are listed in Table A2.

### 4.6. Plasmolysis Assay

The young leaves of *Nicotiana benthamiana* were infected with the *Agrobacterium* strain GV3101, carrying pMDC32-*FvePL1*-GFP vector or empty vector pMDC32-GFP as control, individually. At three days after agroinfiltration, *N. benthamiana* leaves were infiltrated with 10% NaCl solution. Then, leaf sections were excised 10~15 min after injection and examined using confocal laser microscopy (Zeiss, Germany).

### 4.7. Seed Observation

Four-day post-anthesis (DPA) seeds were dissected from achenes and imaged using a Zeiss Stemi SV 6 microscope. For confocal analysis, seeds were fixed in 4% glutaraldehyde (12.5 mM cacodylate, pH 6.9), dehydrated through ethanol gradients (30~100%), cleared in benzyl benzoate–benzyl alcohol (2:1), and visualized on a confocal microscope (Zeiss, Germany) whereas 488 nm excitation was used to image autofluorescence of the seeds for observing fluorescent [16].

### 4.8. RNA Extraction and Quantitative RT-PCR

Total RNA was extracted using a Total RNA extraction Kit (Promega, Madison, WI, USA) and reverse transcribed to cDNA with PrimerScript RT reagent Kit (TaKaRa, Shiga, Japan). The qRT-PCR was performed on a BioRad CFX96 Real-time system with SYBR Green PCR MasterMix (Applied Biosystems, Thermo Scientific, Foster City, CA, USA) under the following conditions: 5 min at 95 °C, followed by 40 cycles of 10 s at 95 °C, 10 s at 55 °C and 30 s at 72 °C, as well as 10 min at 72 °C. The relative expression level was analyzed using a modified 2^−ΔΔCT^ method. For all qRT-qPCRs, *Fveactin* (*FvH4_4g24420*) was used as the internal control. The qRT-PCR experiments were performed from three biological replicates (with three technical repeats).

### 4.9. Pollen Viability Assay

Pollen grains from newly opened flowers of six seedlings of each genotype were collected and then stained with 30 μL 0.3% 3-(4,5)-dimethylthiahiazo(-z-y1)-2,5-di-phenytetrazoliumromide (MTT) solution (0.3 g MTT dissolved in 100 mL phosphate-buffered saline) for 20~30 min to detect pollen viability. Stained samples were mounted on slides, and a cover glass was attached for observation with an Olympus BX-53 microscope.

### 4.10. Cell Wall Imaging Using SEM and TEM

For scanning electron microscopy (SEM) detection, matured pollen grains were collected directly from strawberry stamens and fixed in 2.5% glutaraldehyde. The fixed samples were rinsed three times with 0.1 M phosphate buffer, with each rinse lasting 15 min. For dehydration, the tissues were sequentially immersed in 30%–50%–70%–80%–90%–95%–100%–100% alcohol, spending 15 min in each, and then in isopentyl acetate for 15 min. Regarding drying, the samples were placed in a critical point dryer K850 for the drying process [32]. Subsequently, samples were immobilized on a specimen mount coated with modeling clay and rapidly frozen in liquid nitrogen for about 30 s under vacuum and coated with gold using Ion Sputter MC1000 (Hitachi, Tokyo, Japan) under scanning electron microscopy SU8100 (Hitachi, Tokyo, Japan) [33].

For transmission electron microscopy (TEM) observation, the above samples were fixed with 4% (*w*/*v*) formaldehyde and 2% (*w*/*v*) glutaraldehyde in 0.1 M PBS (pH 7.2) under vacuum (0.6 bar) at 4 °C for 1 h, during which the vacuum was slowly broken three times, then incubated in fresh fixative solution at 4 °C overnight. The samples were washed three times in phosphate buffer, postfixed for 2 h in 1% (*w*/*v*) osmium tetroxide in phosphate buffer at room temperature, rinsed five times for 5 min in PBS, and dehydrated in a graded ethanol series (30 to 100% *v*/*v*) with 1 h incubations in each bath. The samples were then subjected to embedding, polymerization, ultrathin sectioning, staining, and observation under a transmission electron microscopy HT7800 (Hitachi, Tokyo, Japan).

### 4.11. Calcofluor White and Auramine O Staining

Anthers were collected at 10:00 am on the first day post flowering and mounted on slides. After staining with 0.001% auramine O (1 g Auramine O dissolved in 1 mL phosphate-buffered saline) which was added into 1 mL 17% (*m*/*v*) sugar for 15 min, the images were observed at 488 nm excitation and 500 to 570 nm emission. The intine of dissected anthers was stained by calcofluor white and 10% KOH for 1 min with a cover slip and immediately analyzed using a confocal microscope. Calcofluor white was obtained using the excited images at 340~388 nm, and the emission was collected at 400 to 450 nm.

### 4.12. Pollen Germination Assays In Vitro and In Vivo

To observe the pollen tubes in vitro, pollen grains sampled from six seedlings of each genotype were incubated on a pollen germination medium (100 g/L sucrose, 0.02% boric acid, 0.5% agar) in an incubator at 22 °C for 5–6 h in the dark and finally observed under an Olympus BX53 microscope. We calculated the rate of germination according to germinated pollen/total number of pollens × 100% and measured the pollen tube length using ImageJ software (https://imagej.net/ij/ (accessed on 14 October 2025)). For in vivo pollen germination of *F. vesca*, pistils of wild-type emasculated flowers were hand-pollinated with pollen from three genotypes. Notably, the hybrid pistils of *FvePL1*-RNAi were also collected after crosspollination using pollen from YW. One hour after saturated pollination, pistils were collected, fixed in FAA reagent (10% formalin, 80% ethanol, 10% glacial acetic acid) and vacuumed for 1 h and fixed for 10~12 h, softened with 30% NaOH overnight and washed with ddH_2_O. After bleaching with 6% hydrogen peroxide for 0.5 h, pistils were mounted on a microscope slide using a drop of 0.1% decolorized aniline blue solution for at least four hours and carefully pressed with a cover slip to open the pistil longitudinally. Images were observed under the excited at 488 nm, and the emission at 500 to 570 nm.

### 4.13. Drought Stress Treatment, Water Loss and Total Pectin Assay

Ten seedlings of strawberries of different genotypes were placed in a controlled greenhouse maintained at 20~22 °C and 55~68% humidity. Drought treatment was applied by withholding irrigation for 30 days. Post-stress recovery protocols included rehydration via supplemental irrigation at five days under standard growth conditions. Leaves harvested from the soil-grown plants at the same corresponding time were weighed. Water loss was evaluated by determining the decrease in leaf weight. Twenty leaves from three seedlings were used as a biological repeat. Three biological replicates were used for each genotype. Meanwhile, survival rate was calculated by counting the healthy leaves. Determination of pectin content was adopted using the carbazole colorimetric method according to the previous study [15].

### 4.14. Statistical Analysis

All experimental results are presented as mean ± standard deviation (SD) (n ≥ 3). Data were analyzed and visualized using GraphPad Prism (GraphPad software Inc., version 8.0, Boston, MA, USA). The one-way analysis of variance (ANOVA) followed by Tukey’s test was employed to determine the statistical significance (*, *p* < 0.05; **, *p* < 0.01; ***, *p* < 0.001).

## 5. Conclusions

This study comprehensively demonstrated that the downregulation of *FvePL1* resulted in the failures of seed and achene development, and established its essential role in pollen grain exine and intine formation, as well as pollen tube germination. We further identified that suppression of *FvePL1*-induced delayed and incomplete tapetal degeneration, resulting in impaired pollen maturation, reduced pollen number, and decreased viability. In addition, the deficiency of *FvePL1*-enhanced drought tolerance through regulating cell wall structure was determined. These findings provide a mechanistic basis of the function of *FvePL1* in male fertility and stress adaptation, offering potential applications for improving crop yield and resilience under abiotic stress.

## Data Availability

The original contributions presented in this study are included in the article. Further inquiries can be directed to the corresponding author.

## Abbreviations

The following abbreviations are used in this manuscript:

PL	pectate lyase
YW	Yellow Wonder 5AF7
MTT	3-(4,5)-dimethylthiahiazo(-z-y1)-2,5-di-phenytetrazoliumromide
GFP	green fluorescent protein
GDR	Genome database for Rosaceae
DPA	day post-anthesis

## Appendix A

**Table A1:** The list of primers used in this study.

Name	Forward Sequence (5′-3′)	Reverse Sequence (5′-3′)	Application
*FvePL1*	ATGAGGTTAGCTAGCTCGAGG	TTAACATTGACGACCAAACCG	Amplification
pM999-*FvePL1*	GCTCTAGAATGAGGTTAGCTAGCTCGAGG	GCTCTAGAACATTGACGACCAAACCGGCA	Subcellular location
*FvePL1*-RNAi	GGACTAGTGGTACCACGACGACTGGAATGAGCACG	CGAGCTCGGATCCAGGCTTGCAGTCGTGGATGTG	RNAi line
*FvePL1*-OE	CGGGGTACCATGAGGTTAGCTAGCTCGAGG	CGAGCTCTTAACATTGACGACCAAACCG	Overexpression line
q-*FvePL1*	GGAAGTGCTGATCCTACCATTA	CTGTGGTCTGAACTCTATGTGT	qRT-PCR
*Fveactin*	CCCAAGTAAGGATGCCCCCATGTTCG	TTGGCAAGGGGAGCAAGACAGTTGGTAG	qRT-PCR
*FvePL1*	TCTCATGCGCAACAGGGAAT	GTTGCGGCCGAATCCAATAC	ISH
Hyg	ACGTTGCAAGACCTGCCTGAAA	TCCAGTCAATGACCGCTGTTATGC	Positive verification

**Table A2:** The accession number of PL gene family in different species.

Gene ID	Accession Number	Gene ID	Accession Number
OsPLL1	LOC_Os01g36620	AtPLL1	At3g09540
OsPLL2	LOC_Os01g62000	AtPLL2	At3g55140
OsPLL3	LOC_Os02g12300	AtPLL3	At5g09280
OsPLL4	LOC_Os04g05050	AtPLL4	At4g22080
OsPLL5	LOC_Os05g22800	AtPLL5	At4g22090
OsPLL6	LOC_Os06g05209	AtPLL6	At1g11920
OsPLL7	LOC_Os06g05260	AtPLL7	At1g30350
OsPLL8	LOC_Os06g05272	AtPLL8	At1g14420
OsPLL9	LOC_Os06g38510	AtPLL9	At3g02720
OsPLL10	LOC_Os06g38520	AtPLL10	At3g01270
OsPLL11	LOC_Os08g18970	AtPLL11	At5g15110
FvePL1	FvH4_2g19540	AtPLL12	At5g04310
FvePL2	FvH4_3g00620	AtPLL13	At3g54920
FvePL3	FvH4_3g01680	AtPLL14	At5g55720
FvePL4	FvH4_4g05760	AtPLL15	At5g63180
FvePL5	FvH4_4g25110	AtPLL16	At1g67750
FvePL6	FvH4_4g31900	AtPLL17	At3g53190
FvePL7	FvH4_5g06720	AtPLL18	At3g27400
FvePL8	FvH4_5g15430	AtPLL19	At4g24780
FvePL9	FvH4_5g27090	AtPLL20	At3g07010
FvePL10	FvH4_6g02350	AtPLL21	At5g48900
FvePL11	FvH4_6g14680	AtPLL22	At3g24670
FvePL12	FvH4_6g16670	AtPLL23	At4g13210
FvePL13	FvH4_6g49230	AtPLL24	At3g24230
FvePL14	FvH4_6g52190	AtPLL25	At4g13710
FvePL15	FvH4_7g18400	AtPLL26	At1g04680
FvePL16	FvH4_7g20920	SlPL1	Solyc01g010740
SlPL2	Solyc02g067450	SlPL12	Solyc05g014000
SlPL3	Solyc02g080910	SlPL13	Solyc05g055510
SlPL4	Solyc02g087670	SlPL14	Solyc06g071020
SlPL5	Solyc02g093580	SlPL15	Solyc06g071840
SlPL6	Solyc03g058890	SlPL16	Solyc06g083580
SlPL7	Solyc03g058910	SlPL17	Solyc09g005850
SlPL8	Solyc03g071570	SlPL18	Solyc09g008380
SlPL9	Solyc03g113150	SlPL19	Solyc09g061890
SlPL10	Solyc04g010230	SlPL20	Solyc09g091430
SlPL11	Solyc05g007080	SlPL21	Solyc11g008140
